# Supplementary material for: Disruption of CTCF binding by germline non-coding variants in CDKN2B suppress CDKN2A expression and predispose to melanoma
Source: medRxiv. 2026 Jun 2:2026.06.01.26352322. Preprint. [Version 1] doi: 10.64898/2026.06.01.26352322 (PMC13252464; doi:10.64898/2026.06.01.26352322)
Supplement: Supplementary file 1 [file NIHPP2026.06.01.26352322v1-supplement-2.pdf]

medRxiv preprint doi: https://doi.org/10.64898/2026.06.01.26352322; this version posted June 2, 2026. The copyright holder for this preprint (which was not certified by peer review) is the author/funder, who has granted medRxiv a license to display the preprint in perpetuity. This article is a US Government work. It is not subject to copyright under 17 USC 105 and is also made available for use under a CC0 license.

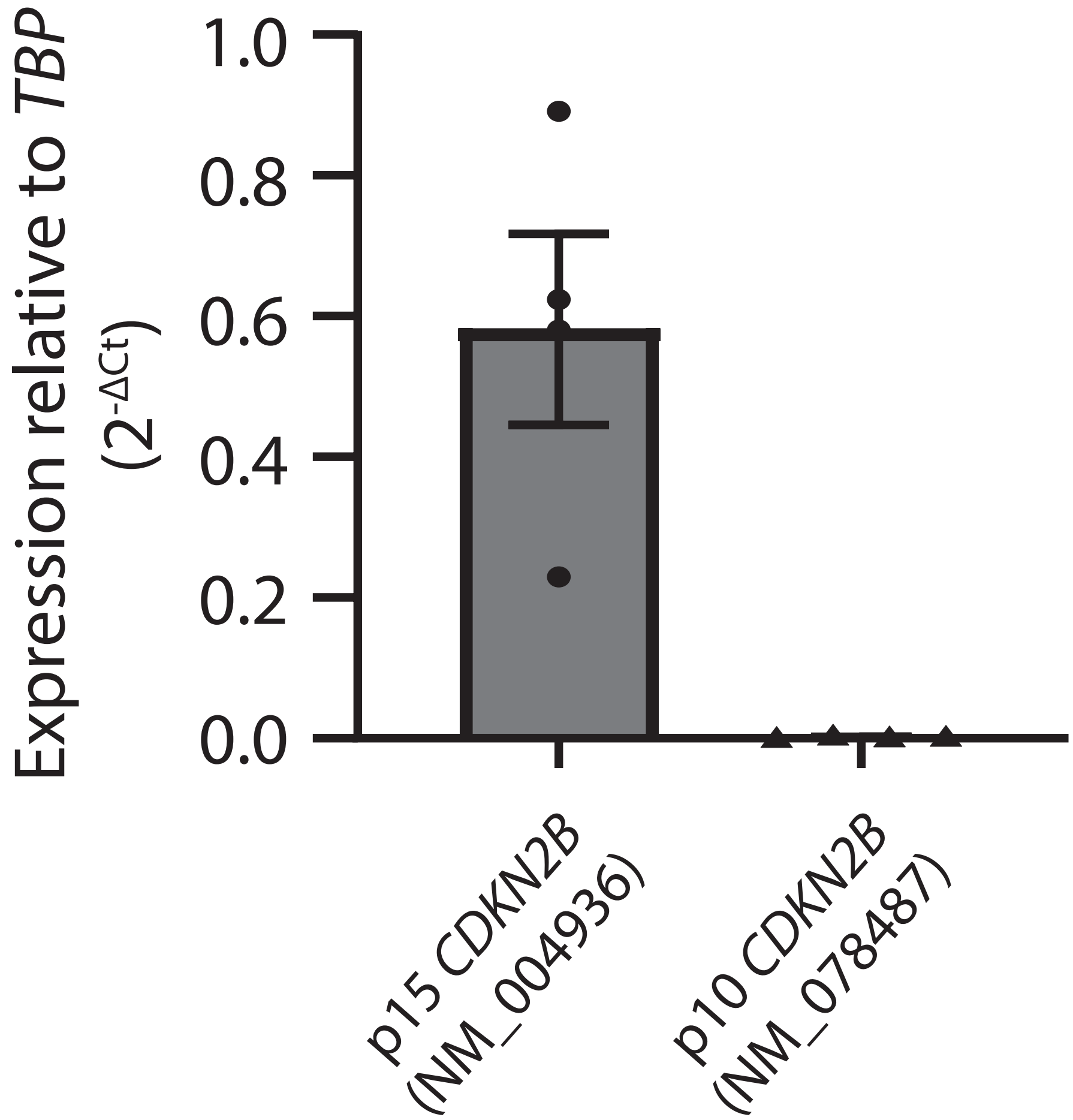

Supplementary Figure 1. Expression of *CDKN2B* p15 and p10 isoforms in immortalized human melanocytes. *CDKN2B* transcript expression was assessed by isoform-specific TaqMan qRT-PCR assays normalized relative to expression of TBP. Each data point shows the average of three technical replicates for four biological replicates.

medRxiv preprint doi: https://doi.org/10.64898/2026.06.01.26352322; this version posted June 2, 2026. The copyright holder for this preprint (which was not certified by peer review) is the author/funder, who has granted medRxiv a license to display the preprint in perpetuity.
This article is a US Government work. It is not subject to copyright under 17 USC 105 and is also made available for use under a CC0 license.

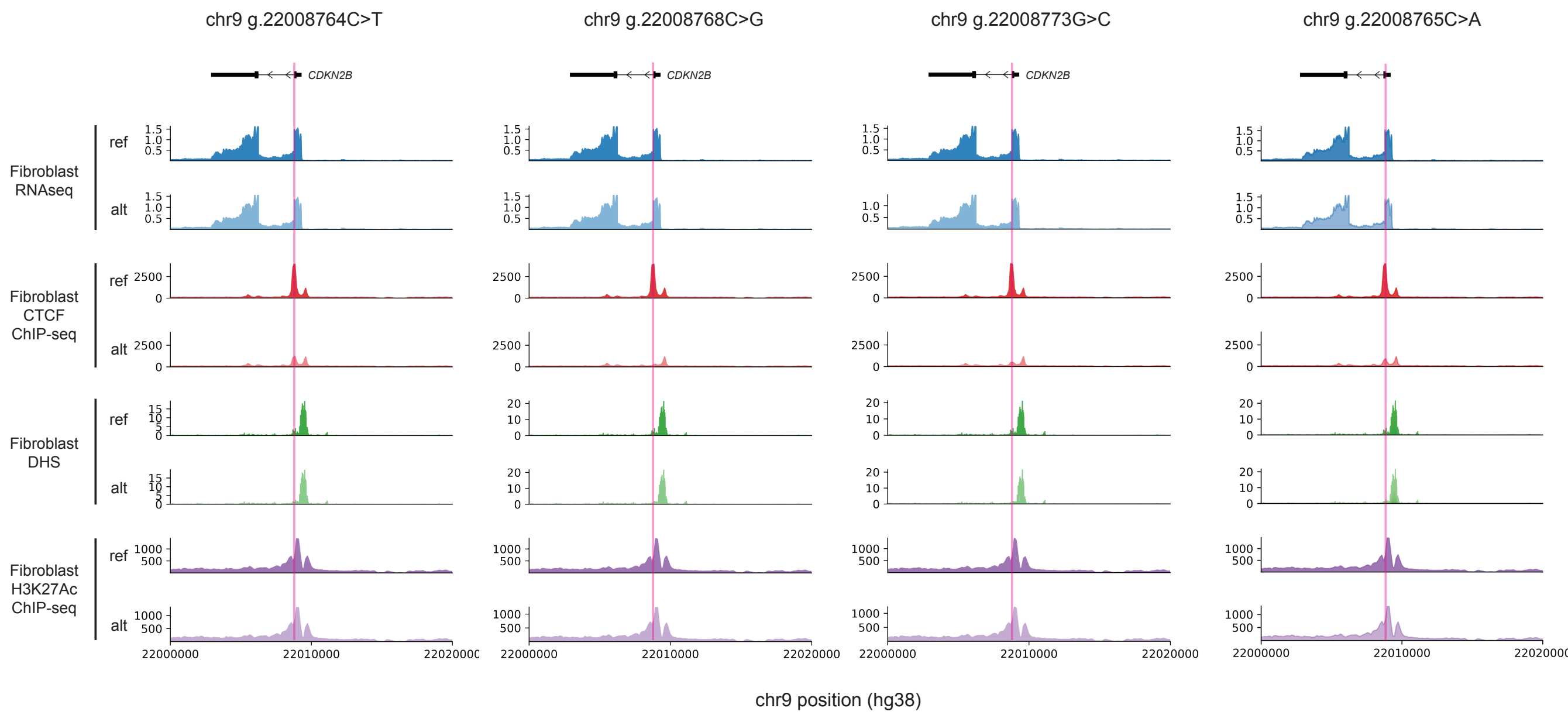

Supplementary Figure 2. *CDKN2B* variants are predicted to alter CTCF binding and chromatin accessibility. AlphaGenome predictions of the consequences of each of the variants identified in American melanoma cases and UKBB on transcription (RNA-seq), CTCF-binding (CTCF ChIP-seq), chromatin accessibility as assessed by DNAseI hypersensitivity sequencing (DHS), and histone mark H3K27Ac (H3K27Ac ChIP-seq). The predictions suggest predicted loss of CTCF binding as well as a modest reduction of chromatin accessibility over each of the variant positions for alternative alleles (alt) relative to the reference (ref). Variant position is highlighted in pink; genes in black at the top of each panel.

medRxiv preprint doi: https://doi.org/10.64898/2026.06.01.26352322; this version posted June 2, 2026. The copyright holder for this preprint (which was not certified by peer review) is the author/funder, who has granted medRxiv a license to display the preprint in perpetuity.
This article is a US Government work. It is not subject to copyright under 17 USC 105 and is also made available for use under a CC0 license.

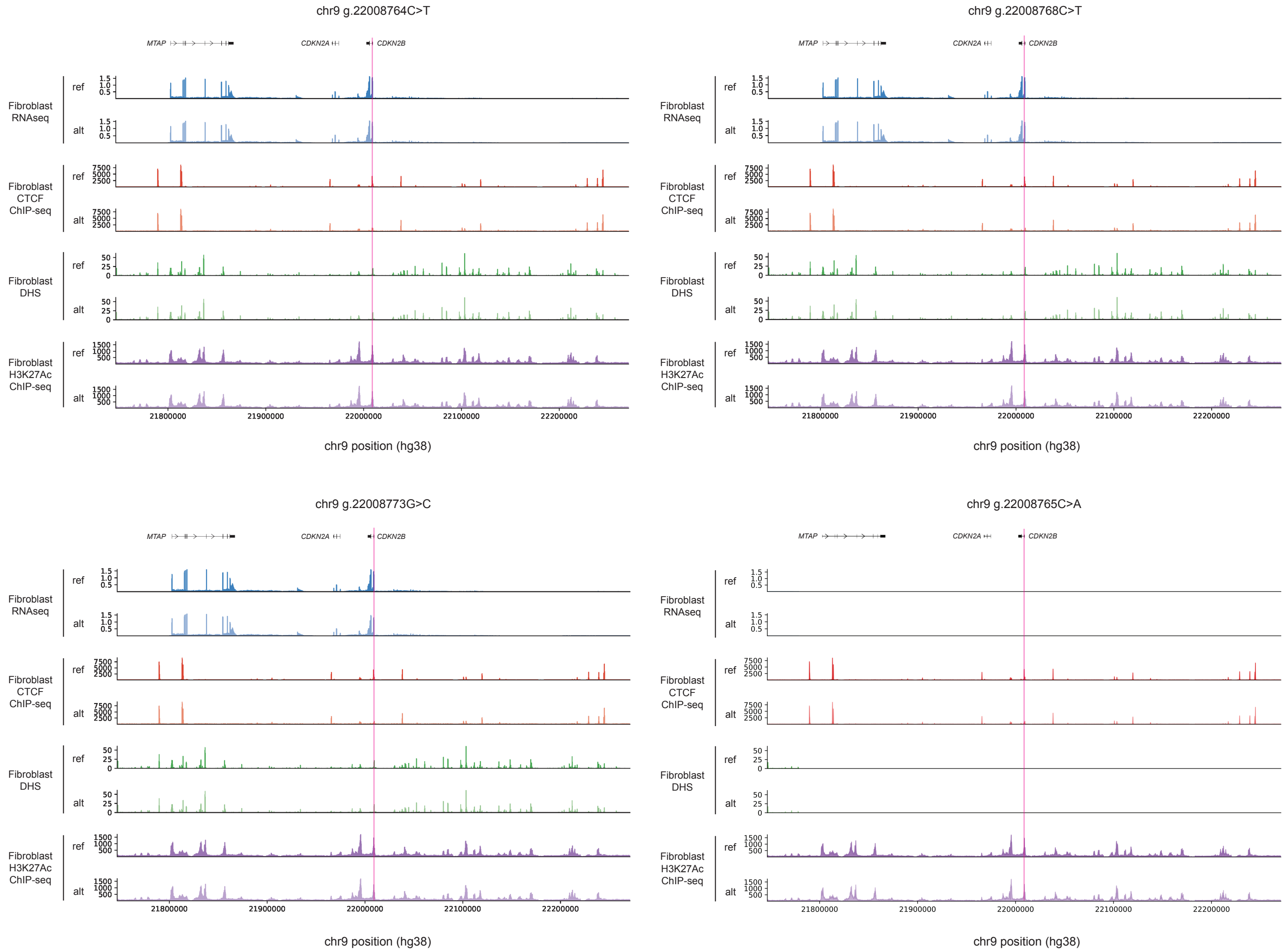

Supplementary Figure 3. *CDKN2B* variants are predicted to alter CTCF binding and chromatin accessibility. A zoomed-out view of AlphaGenome predictions of the consequences of each of the variants identified in American melanoma cases and UKBB on transcription (RNA-seq), CTCF-binding (CTCF ChIP-seq), chromatin accessibility as assessed by DNAseI hypersensitivity sequencing (DHS), and histone mark H3K27Ac (H3K27Ac ChIP-seq). The predictions suggest predicted loss of CTCF binding as well as a modest reduction of chromatin accessibility over each of the variant positions for alternative alleles (alt) relative to the reference (ref). Variant position is highlighted in pink; genes in black at the top of each panel.

medRxiv preprint doi: https://doi.org/10.64898/2026.06.01.26352322; this version posted June 2, 2026. The copyright holder for this preprint (which was not certified by peer review) is the author/funder, who has granted medRxiv a license to display the preprint in perpetuity.
This article is a US Government work. It is not subject to copyright under 17 USC 105 and is also made available for use under a CC0 license.

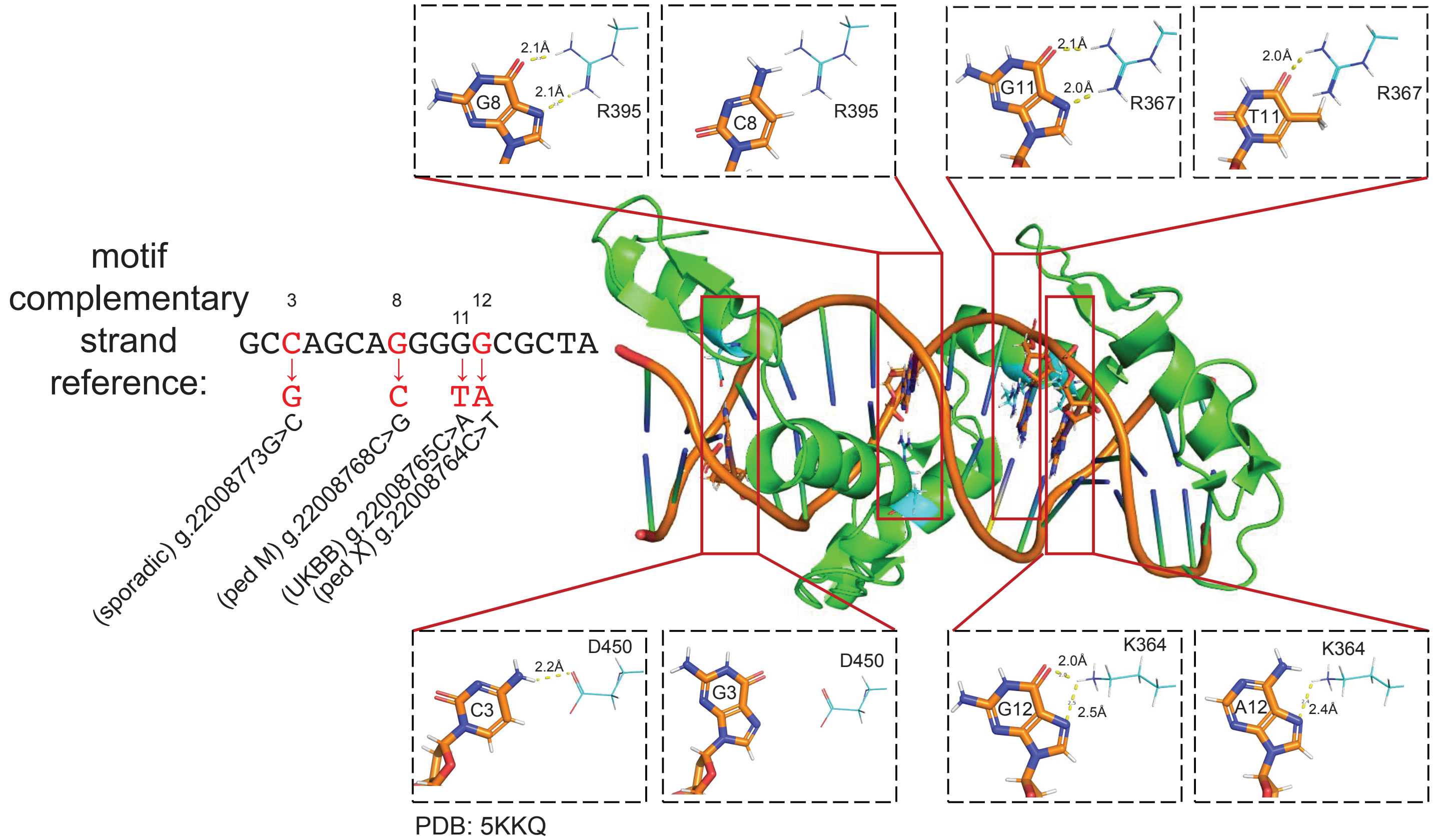

Supplementary Figure 4. Melanoma risk variants alter CTCF interaction with DNA. Cytosine at position 3 (g.22008773G), guanine at position 8 (g.22008768C), guanine at position 11 (g22008765C), and guanine at position 12 (g.22008764C) in the complementary strand of the CTCF DNA-binding motif form hydrogen bonds (yellow dotted line) with D450, R395, R367, and K364 of the CTCF protein, respectively. These are lost upon their respective base substitutions observed in melanoma cases. Structures are generated from PDB:5KKQ. The minus-strand of the CTCF-binding site is shown and labeled with respective base substitutions.

medRxiv preprint doi: https://doi.org/10.64898/2026.06.01.26352322; this version posted June 2, 2026. The copyright holder for this preprint (which was not certified by peer review) is the author/funder, who has granted medRxiv a license to display the preprint in perpetuity. This article is a US Government work. It is not subject to copyright under 17 USC 105 and is also made available for use under a CC0 license.

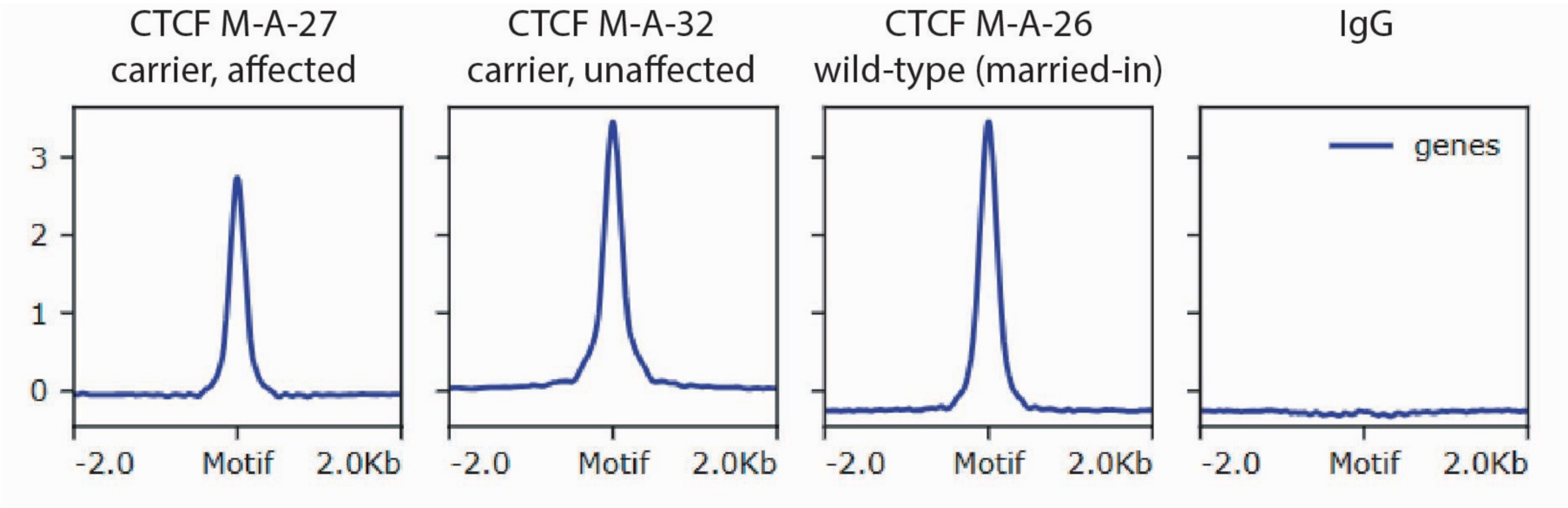

Supplementary Figure 5. CTCF ChIP seq assessment of fibroblasts from carriers of familial sequence variants. Peak enrichment over known CTCF binding sites is shown by CTCF ChIP-seq experiments from fibroblasts from two carriers (M-A-27 and M-A-37) and one non-carrier (M-A-26).

medRxiv preprint doi: https://doi.org/10.64898/2026.06.01.26352322; this version posted June 2, 2026. The copyright holder for this preprint (which was not certified by peer review) is the author/funder, who has granted medRxiv a license to display the preprint in perpetuity. This article is a US Government work. It is not subject to copyright under 17 USC 105 and is also made available for use under a CC0 license.

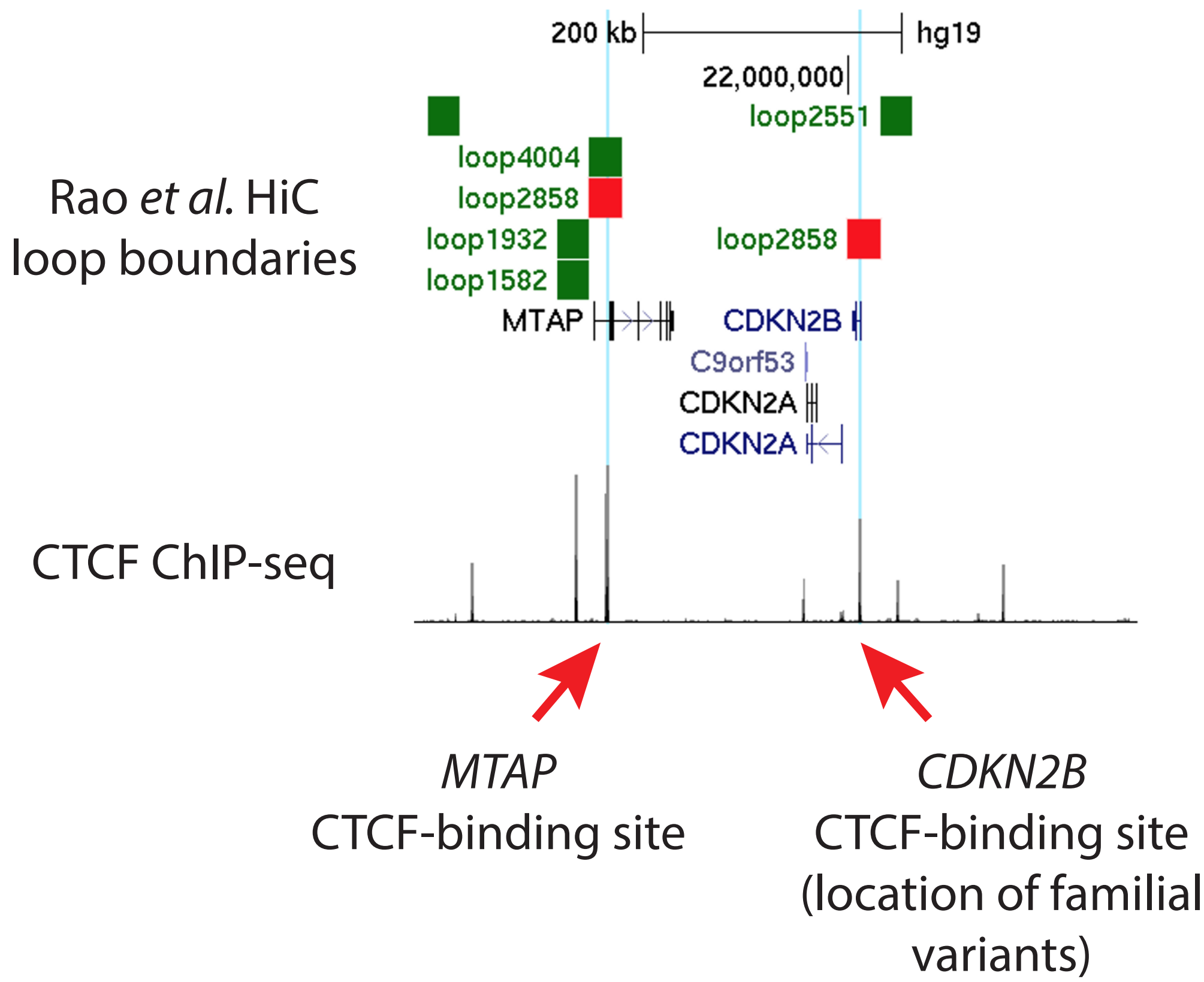

Supplementary Figure 6. UCSC Genome Browser representation of CTCF ChIP coverage as signal normalized to input and HiC loops identified by Rao and colleagues (upper). The Rao loop that covers the *CDKN2B* CTCF binding site (ID loop 2858) is highlighted in red. The *CDKN2B* CTCF binding site that contains mutations is highlighted in blue and indicated with a red arrow. The MTAP CTCF binding site that forms a loop with *CDKN2B* one is also highlighted in blue and indicated with a red arrow. Scale bar indicating length equivalent to 200kb is given at the top.

medRxiv preprint doi: https://doi.org/10.64898/2026.06.01.26352322; this version posted June 2, 2026. The copyright holder for this preprint
(which was not certified by peer review) is the author/funder, who has granted medRxiv a license to display the preprint in perpetuity.
This article is a US Government work. It is not subject to copyright under 17 USC 105 and is also made available
for use under a CC0 license.

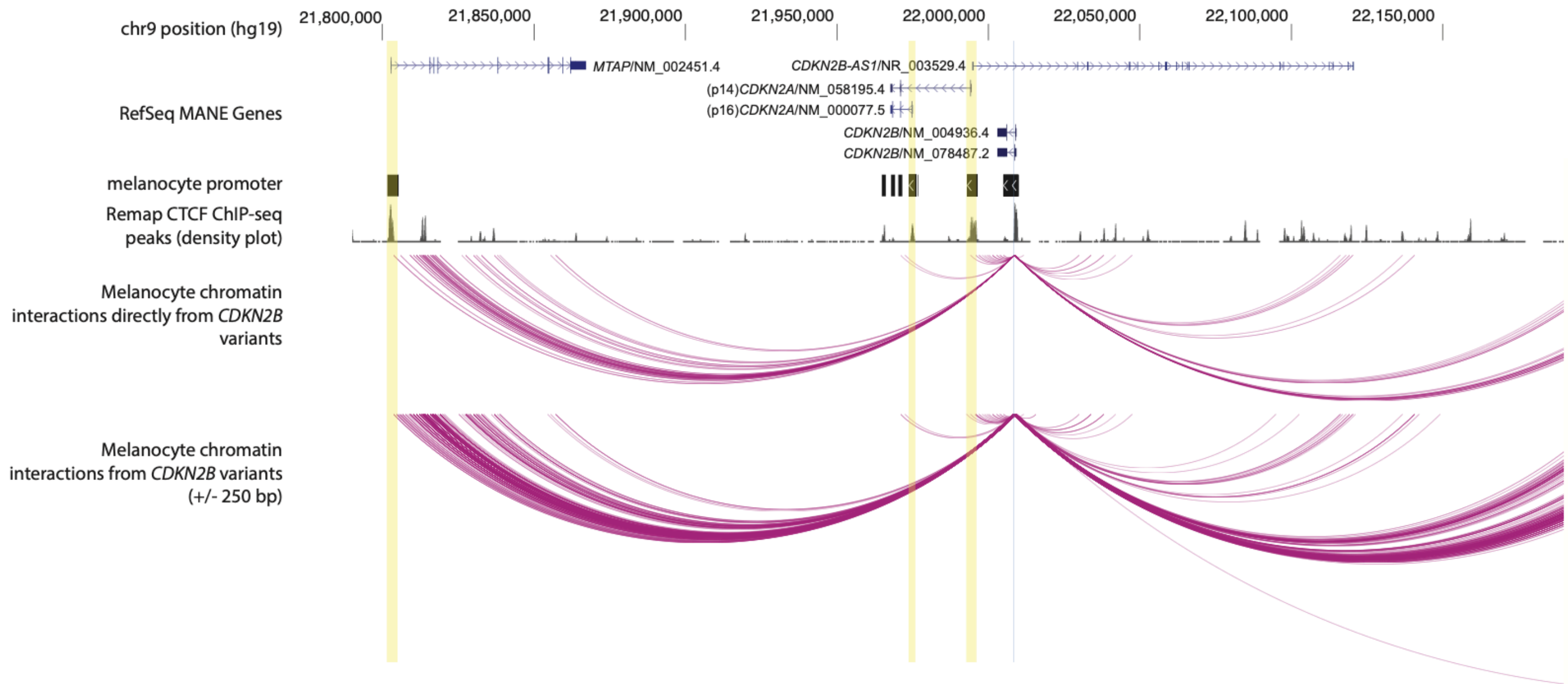

Supplementary Figure 7. Chromatin interactions with the *CDKN2B* variant region. Chromatin interactions from the region harboring melanoma risk-associated *CDKN2B* variants in human primary melanocytes. Data are from a previously published capture-HiC assay from Thakur and colleagues. Interactions were assessed using capture-baits tiled across the 9p21 region. Data are filtered to show only the most highly-scored (80th percentile or higher of significant interactions) interactions directly from the region harboring the variants (upper panel, highlighted in light blue) or from a larger 500 bp window surrounding the variants (lower panel). Annotated melanocyte promoter regions (ChromHMM) from the Roadmap Epigenome Project are shown, as are the density of annotated ENCODE CTCF-binding sites from ReMap. Promoters of the p16 and p14 transcripts of *CDKN2A*, as well as the promoter of MTAP, are highlighted in yellow. Coordinates are hg38.

medRxiv preprint doi: https://doi.org/10.64898/2026.06.01.26352322; this version posted June 2, 2026. The copyright holder for this preprint (which was not certified by peer review) is the author/funder, who has granted medRxiv a license to display the preprint in perpetuity.
This article is a US Government work. It is not subject to copyright under 17 USC 105 and is also made available for use under a CC0 license.

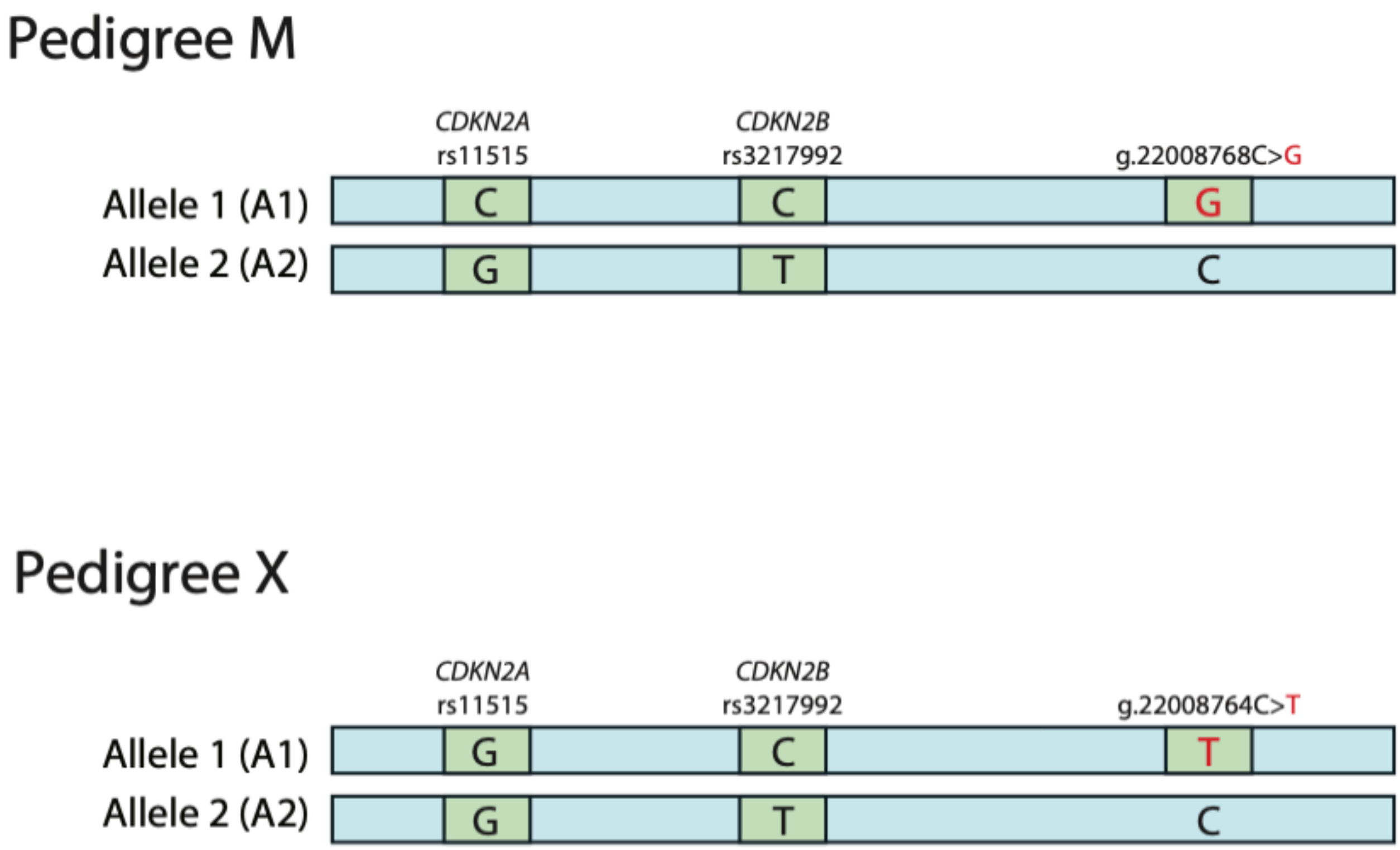

Supplementary Figure 8. Schematic of SNP allele phasing relative to familial *CDKN2B* variants. Phase of variants for patient-derived samples from melanoma families was assessed via long-read whole-genome sequencing (see **Methods**).

medRxiv preprint doi: https://doi.org/10.64898/2026.06.01.26352322; this version posted June 2, 2026. The copyright holder for this preprint (which was not certified by peer review) is the author/funder, who has granted medRxiv a license to display the preprint in perpetuity. This article is a US Government work. It is not subject to copyright under 17 USC 105 and is also made available for use under a CC0 license.

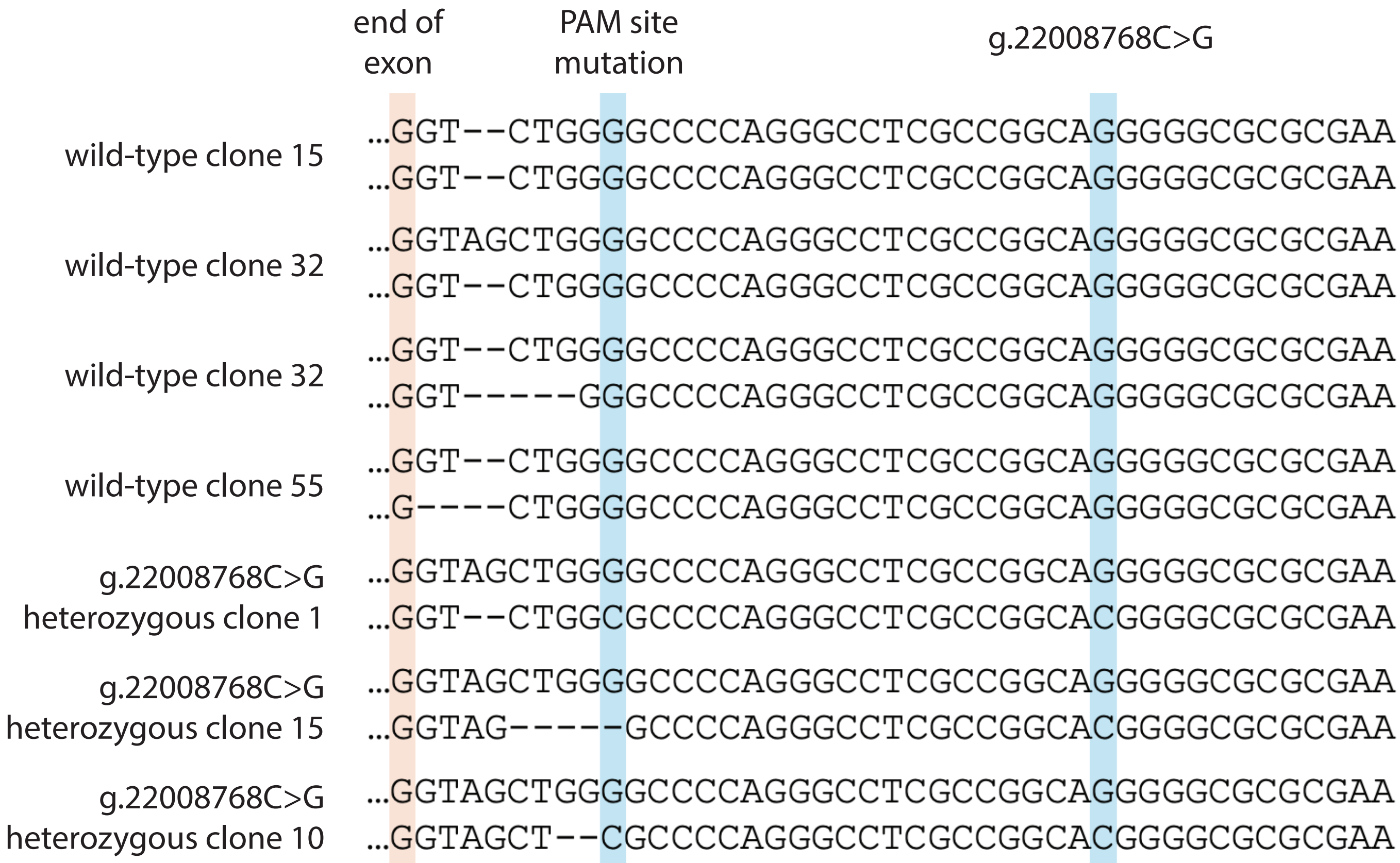

Supplementary Figure 9. DNA sequence deconvolution for CRISPR-Cas9-edited primary melanocytes. Each of two alleles for each clone as assessed via Sanger sequencing. Sequence is shown relative to the direction of the *CDKN2B* transcript (minus strand of the genome); a successful edit for the g.22008768C>G variant is shown as a “C” allele. The relative sites of the end of the first exon of the CDKN2B p15 transcript and engineered PAM site mutation are shown. Deleted based are denoted by “-“.

medRxiv preprint doi: https://doi.org/10.64898/2026.06.01.26352322; this version posted June 2, 2026. The copyright holder for this preprint (which was not certified by peer review) is the author/funder, who has granted medRxiv a license to display the preprint in perpetuity.
This article is a US Government work. It is not subject to copyright under 17 USC 105 and is also made available for use under a CC0 license.

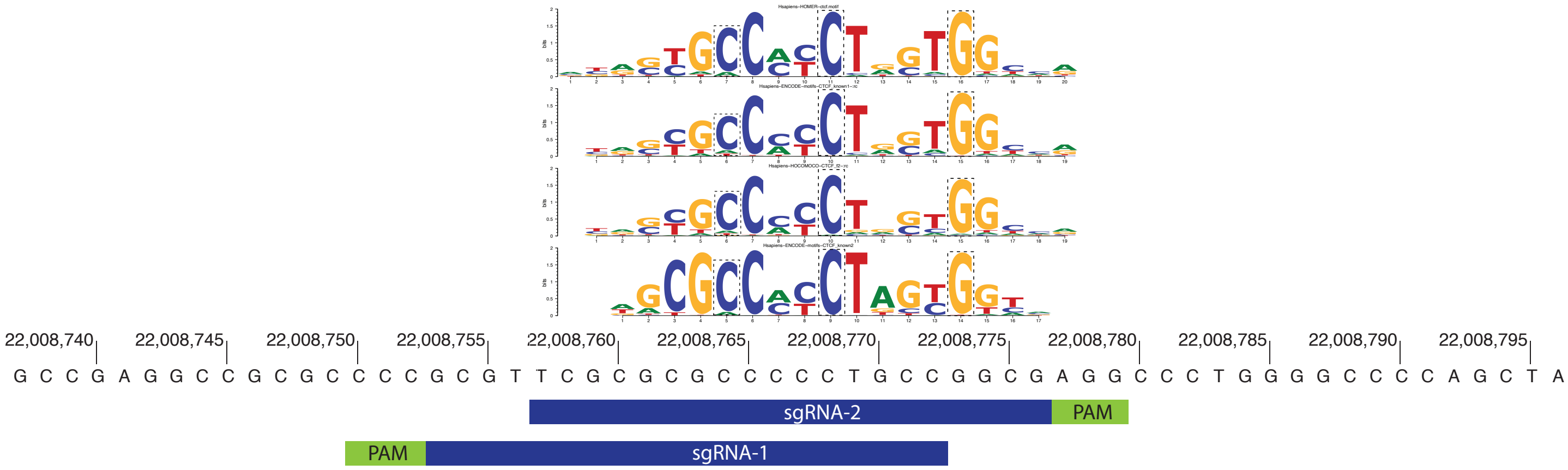

Supplementary Figure 10. Location of small guide RNAs (sgRNAs) used for CRISPR-inhibition experiments. Locations of guides are shown relative to the CTCF motif. Locations of the three risk-associated variants is denoted on the motif by based outlined in a dashed rectangle.
